# Transcriptome analysis provides new insights into cold adaptation of corsac fox (*Vulpes Corsac*)

**DOI:** 10.1002/ece3.8866

**Published:** 2022-04-19

**Authors:** Xiufeng Yang, Guolei Sun, Tian Xia, Muha Cha, Lei Zhang, Bo Pang, Qingming Tang, Huashan Dou, Honghai Zhang

**Affiliations:** ^1^ 56650 College of Life Science Qufu Normal University Qufu China; ^2^ Hulunbuir Academy of Inland Lakes in Northern Cold & Arid Areas Hulunbuir China; ^3^ Hulun Buir Forestry and Grassland Business Development Center Hulunbuir China

**Keywords:** cold adaptation, gene tree discordance, selective pressure analysis, transcriptome, *Vulpes corsac*

## Abstract

*Vulpes*are widely distributed throughout the world and have undergone drastic physiological and phenotypic changes in response to their environment. However, little is known about the underlying genetic causes of these traits, especially *Vulpes corsac*. In this study, RNA‐Seq was used to obtain a comprehensive dataset for multiple pooled tissues of corsac fox, and selection analysis of orthologous genes was performed to identify the genes that may be influenced by the low‐temperature environment. More than 6.32 Gb clean reads were obtained and assembled into a total of 173,353 unigenes with an average length of 557 bp for corsac fox. Selective pressure analysis showed that 16 positively selected genes (PSGs) were identified in corsac fox, red fox, and arctic fox. Enrichment analysis of PSGs showed that the *LRP11* gene was enriched in several pathways related to the low‐temperature response and might play a key role in response to environmental stimuli of foxes. In addition, several positively selected genes were related to DNA damage repair (*ELP2* and *CHAF1A*), innate immunity (*ARRDC4* and *S100A12*), and the respiratory chain (*NDUFA5*), and these positively selected genes might play a role in adaptation to harsh wild fox environments. The results of common orthologous gene analysis showed that gene flow or convergent evolution might be an important factor in promoting regional differentiation of foxes. Our study provides a valuable transcriptomic resource for the evolutionary history of the corsac fox and the adaptations to the extreme environments.

## Graphical abstract

Assembled and characterized the first transcriptome data for the corsac fox using RNA‐Seq technology and revealed candidate genes LRP11 and several pathway that might be involved in cold environment adaption in foxes.

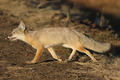

## 1 INTRODUCTION

Adaptation is a process in which organisms adapt to their environment to survive and reproduce in terms of morphological structures and physiological functions (Brandon, 1978). In particular, creatures in extreme environments have undergone adaptive evolution in phenotype, physiology, and behavior, and the study of its adaptive mechanism has always been a hot topic in field of evolutionary biology. Numerous studies have explored in depth the mechanisms of adaptation in different species at the genomic, metagenomic, and transcriptome levels, such as sheep (Ji et al., 2016), yak (Lan et al., 2021), goat (Martchenko et al., 2020; Zhang et al., 2021), and dog (Gou et al., 2014; Miao et al., 2017; Wu et al., 2016).

As a typical example of adaptation, the fox distributed widely and the population remains stable around the world (IUCN, 2021, Figure 1). The members of this genus play important roles in maintaining ecosystems, including tundra, forests, deserts, and grasslands (Statham et al., 2014). Accordingly, they have undergone adaptive evolution in physiology, morphology, and other aspects. However, little is currently known about their underlying genetic causes, something we aimed to explore in this study. Research on the molecular ecology of the *Vulpes* has mainly focused on red fox and arctic fox, while corsac fox currently exhibits only in behavior, diet, and zoonoses (Abdybekova et al., 2015; Guilherme et al., 1938; Lkhagvasuren et al., 2016). In the study of adaptive evolution, genome‐wide extension of unique gene families and positively selected genes potentially play roles in the adaptation of extreme environments in arctic fox (Peng et al., 2021). Transcriptome analysis of arctics and the red fox has shown that fatty acid metabolism genes are under positive selection in the arctic fox and seem generally to be crucial for mammalian survival under arctic conditions (Kumar et al., 2015). In our previous study, several positively selected genes related to natural immunity (*CFI* and *LRRFIP1*), protein synthesis (*GOLGA4*, *CEP19*, and *SLC35A2*), and DNA damage repair (*MDC1*) were screened in farmed fox, which might play a role in adaptation to breeding environments (Yang et al., 2021).

**FIGURE 1:**
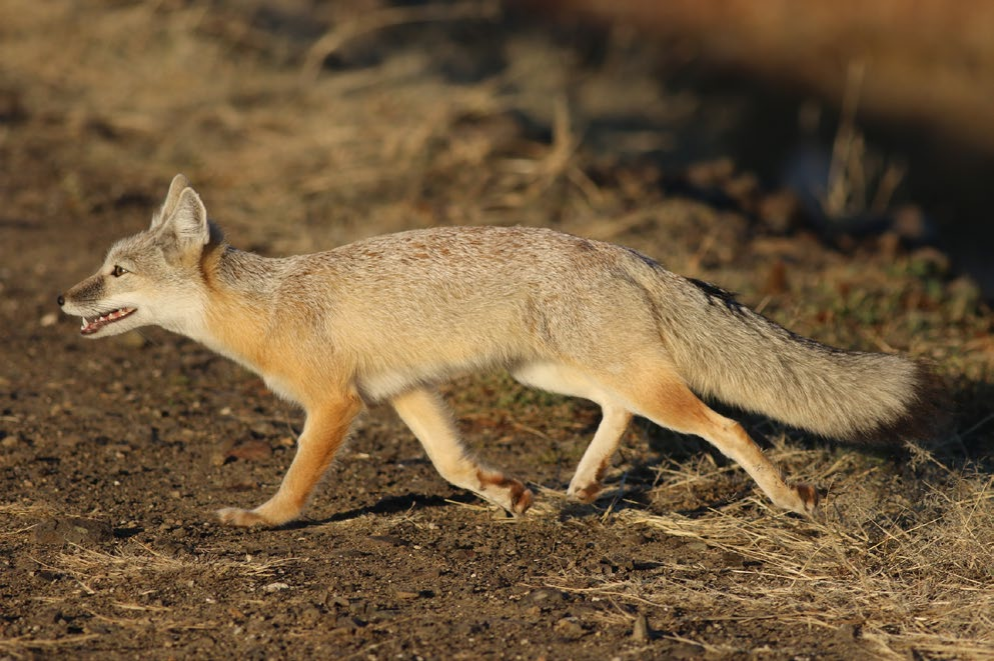
Corsac fox in HulunBuir grassland, Inner Mongolia

Transcriptome analysis is an economic strategy of functional genomic studies that enables the collection of gene sequences for nonmodel animals. These advances greatly facilitate comparative evolutionary studies (Emrich et al., 2007; Garber et al., 2011; Zhang et al., 2010). Despite not representing an entire genome, this transcriptome analysis identified numerous genes that are relevant to adaptation in species. Transcriptome analysis has been used for the assembly and annotation of transcriptomes in many Canidae, including wolves (Liu et al., 2017, 2019), dogs (Yang et al., 2018), foxes (Kukekova et al., 2011; Kumar et al., 2015; Yang et al., 2021), and Raccoon dogs (Du et al., 2017). These studies have identified many genes involved in adaptive evolution and have contributed to the development of extensive genomic and transcriptomic resources for Canidae. To date, few studies have investigated wild environment adaptation in corsac fox, and the molecular mechanisms have not yet been illuminated. In this study, the pooled transcriptome of multiple tissues of corsac fox was sequenced and compared with red fox and arctic fox to explore the molecular mechanism and corresponding genes of their adaptation to cold environments. The findings provide insights into the molecular mechanisms of adaptation to extreme environments in *Vulpes*.

## 2 MATERIALS AND METHODS

### 2.1 Sample collection

Kidney, brain, and liver tissue of one *Vulpes corsac* (corsac fox, named CF) was collected from Hulun Lake National Nature Reserve, Inner Mongolia, China. The tissue sample was stored in RNAlater RNA stabilization reagent (QIAGEN, USA) and frozen at −80℃ in ultradeep‐freeze equipment. The data of two *vulpes lagopus* (arctic fox, named AF1 and AF2) and one *vulpes vulpes* (red fox, named RF) were downloaded from the National Center for Biotechnology Information (NCBI) Sequence Read Archive (SRA) database with accession numbers ERR687853, ERR687854, and ERR687855, respectively, and this sample was collected from Kronoby, Finland (Kumar et al., 2015).

### 2.2 RNA extraction, library construction, and sequencing

Total RNA of CF was extracted using RNeasy Mini Kit (QIAGEN, USA) and checked for purity using 1% agarose gels and a NanoPhotometer^®^ spectrophotometer (IMPLEN, USA). The integrity and concentration of RNA were measured and assessed using the RNA Nano 6000 Assay Kit of the Agilent Bioanalyzer 2100 system (Agilent Technologies, USA) and the Qubit^®^ RNA Assay Kit (Invitrogen, USA) in a Qubit^®^ 2.0 Flurometer (Life Technologies, USA), respectively. Total RNA (at least 3 mg per tissue) was used to construct sequencing libraries with the NEBNext^®^ Ultra™ RNA Library Prep Kit for Illumina^®^ (NEB, USA). Once the library was built, an Agilent Bioanalyzer 2100 system was used to assess the library quality and then sequenced on an Illumina HiSeq 2500 platform.

### 2.3 Transcriptome assembly, gene functional annotation, and CDS identification

Clean reads were obtained from raw reads by removing reads that contained adapter or poly‐N and low‐quality reads (*Q*‐value ≤ 20) (Cock et al., 2010). Transcriptome assembly was carried out with Trinity (Grabherr et al., 2011) to obtain reference transcriptome for subsequent analysis. Annotation of gene functional included pathway annotation, protein functions annotation, Gene Ontology (GO) annotation, and COG/KOG functional annotation. The website and parameters of those databases are listed in Table S1. The NR and Swiss‐Prot databases were used to blast the CDS of each putative unigene with a cut‐off of 1e‐5, and Estscan software was used to determine the unigene that did not have aligned results (Iseli et al., 1999).

### 2.4 Identification of gene orthologous groups and calculation of Ka/Ks

OrthoMCL (Li et al., 2003) software was used to identify orthologous groups with *Ailuropoda melanoleuca* (aml) and *Canis lupus familiaris* (cfa) as the outside and internal reference genomes, respectively. Sequence and optimized protein alignment results were constructed by Muscle 3.8.3313 and Gblocks_0.91b (Edgar, 2004), respectively. The online software KOBAS (http://kobas.cbi.pku.edu.cn/), DAVID (https://david.ncifcrf.gov/tools.jsp), and the R package ClusterProfiler (Yu et al., 2012) were used for GO and KEGG enrichment analysis. The results of sequence alignment were used to construct evolutionary trees by Phyml (version: 20131016) (Guindon et al., 2005).

The ratio (*ω*) of the number of nonsynonymous substitutions per nonsynonymous site (Ka) to the number of synonymous substitutions per synonymous site (Ks) could be used as an indicator of selective pressure acting on a protein‐coding gene in genetics. When *ω* > 1 was usually said to be evolving under positive selection, *ω* < 1 was usually said to be under purifying selection of homologous genes. In this study, the value of ω was calculated for the pairwise alignments by the Paml‐codeml 4.7 (Yang, 2007) package with default settings.

### 2.5 Homologous gene analysis of three species of fox

The orthologous genes obtained in this study were compared with those screened in previous studies on silver fox and blue fox to acquire the orthologous genes shared by five samples (corsac fox, red fox, arctic fox, blue fox, and silver fox) (Yang et al., 2021). Then, phylogenetic tree was constructed based on orthologous genes and 12 protein‐coding genes (except *ND6*) of mitochondria downloaded from the NCBI database by three methods (PAUP (Swofford, 1998), Bayes (Fredrik & Huelsenbeck, 2003) and RAxML (Alexandros, 2014)), and the reasons for the differences were discussed based on previous studies (Degnan & Rosenberg, 2009; Nakhleh, 2013).

## 3 RESULTS

### 3.1 Overview of transcriptome sequencing data and *de novo* assembly

One cDNA library was constructed from pooled RNA extracts from different tissues (liver, brain, and kidney) of *Vulpes corsac* (CF). The summary statistics of the RNA‐Seq data are shown in Table 1. After filtering the raw data, a total of 73.37 million clean reads were obtained with an average read length of 150 bp base pairs for further analysis. The Q30% and Q20% of the CF sample were 94.13% and 97.69%, respectively. A total of 234.9 million clean reads remained from RF, AF1, and AF2, which were downloaded from the NCBI database.

**TABLE 1:** Summary of sequencing results

Sample	Raw reads	Clean reads	Clean bases (Gb)	Error (%)	Q20 (%)	Q30 (%)	GC (%)
CF	76,627,518	73,370,220	11.01	0.01	97.69	94.13	50.99
RF^a^	87,641,852	81,623,324	7.67	0.08	93.82	84.17	50.21
AF1^a^	72,243,730	67,227,516	6.32	0.09	93.45	83.75	50.39
AF2^a^	92,135,172	86,128,572	8.1	0.08	93.95	84.33	49.84

Abbreviations: AF, arctic fox; CF, corsac fox; RF, red fox.

^a^ The data which download from NCBI database. Error: sequencing error rate. Q20/Q30: Percentage of bases with a Phred value of at least 20/30. GC: The content of G and C.

### 3.2 Function annotation and CDS identification

After assembly, we obtained 555,949 transcripts with a mean length range from 738 to 836 bp and 463,335 unigenes with a mean length range from 557 to 690 bp for the four samples. The N50 values for transcripts and unigenes ranged from 1497 to 1765 and 758 to 1352, respectively. Most of the transcripts and unigenes were distributed within the range of 200–500 bp, which comprised 60.3%–69.3% of the transcripts and 66.3%–76.6% of the unigenes of the whole dataset. For the rest, 8,022–12,876 unigenes had lengths of 1–2 kbp and 4984–10,548 were within >2 kbp. The length distributions of the transcripts and unigenes are shown in Table 2. This large number of reads with paired‐end information produced much longer unigenes (mean: 618 bp) than those in previous studies (Novaes et al., 2008; Parchman et al., 2010).

**TABLE 2:** Length interval and distribution of transcripts and unigenes

Length interval & distribution	Transcripts				Unigenes			
	CF	RF	AF1	AF2	CF	RF	AF1	AF2
200–500 bp	184,053	59,822	52,954	64,584	173,353	53,894	47,236	58,544
500–1 kbp	37,021	16,303	15,419	16,436	29,632	12,367	11,671	12,416
1k‐2 kbp	21,320	12,073	11,054	12,436	12,876	8,022	7,296	8,120
>2 kbp	23,138	9,391	8,347	11,598	10,548	5,617	4,984	6,759
Total Number	265,532	97,589	87,774	105,054	226,409	79,900	71,187	85,839
Mean length	738	786	836	787	557	667	672	690
N50	1,596	1,532	1,765	1,497	758	1,196	1,185	1,352
N90	262	277	280	281	243	252	255	251
Total Nucleotides	195,990,946	76,748,930	87,804,175	69,105,503	126,079,486	53,321,702	47,842,732	59,207,490

Note

N50/N90: The transcript obtained by splicing was arranged from long to short and then accumulated. When the cumulative length>=50%/90% of the total length, then the transcript length is considered N50/N90.

**FIGURE 2:**
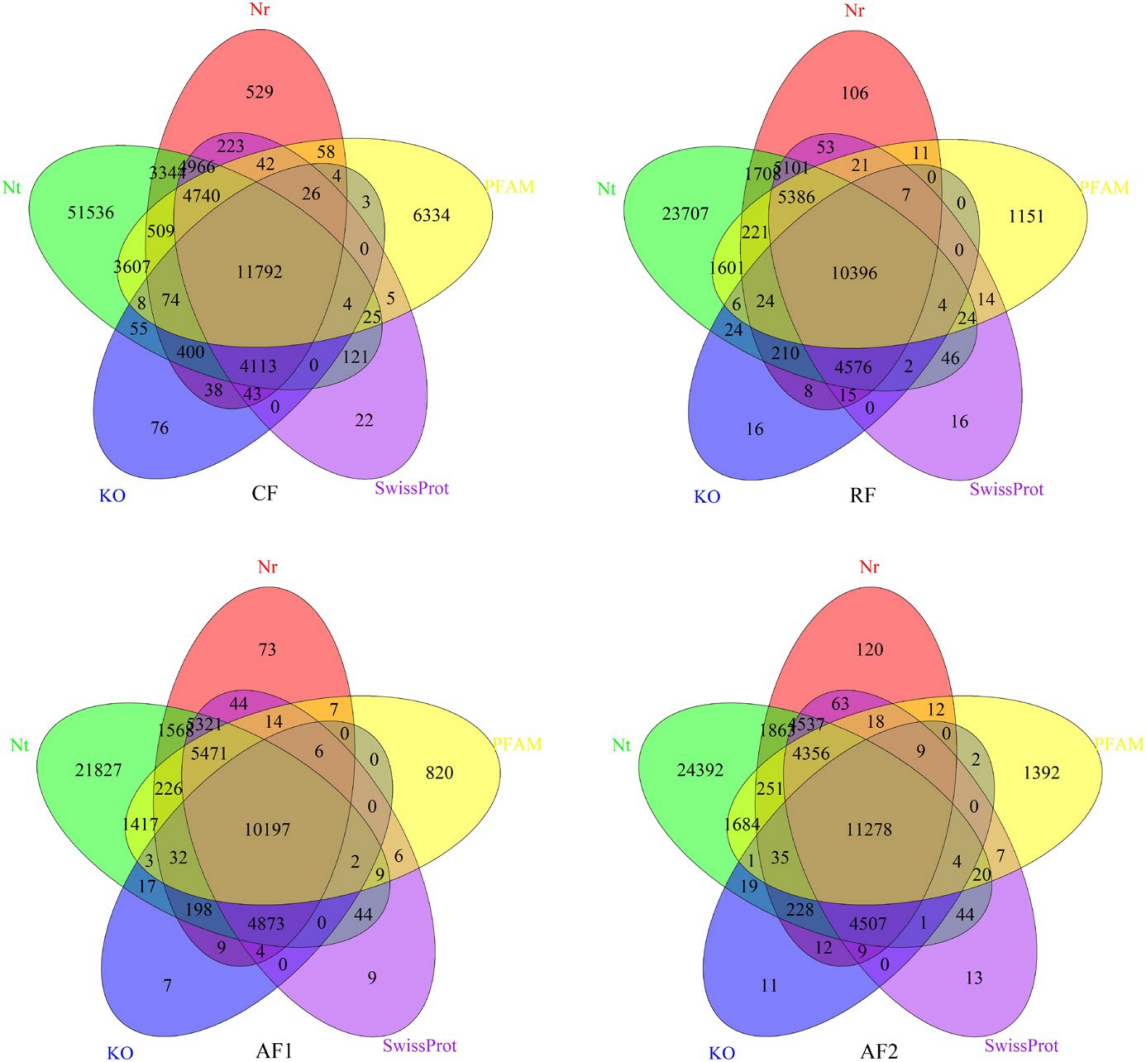
Venn diagram of annotation results from top five databases. Number of annotated unigenes were marked in each circle

To better understand the functional characterization of all assembled unigenes, BLASTX and BLASTN were employed to against five public databases (Nr, Nt, KO, Swiss‐Prot, PFAM, GO, and KOG). A total of 92,697 (40.94%), 54,455 (68.15%), 52,204 (73.33%), and 54,888 (63.94%) unigenes were annotated in at least one database in CF, RF, AF1, and AF1, respectively (Table 3 and Figure 2). Specifically, the Nt database contributed the largest number of matches (CF: 30,901, RF: 27,843, AF1: 28,043, and AF2: 27,298). Furthermore, 5926 (2.62%), 5365 (6.71%), 5328 (7.48%), and 5803 (6.76%) annotated in all databases in CF, RF, AF1, and AF1, respectively.

**TABLE 3:** Statistics of blast results for Unigene against databases

Database	CF	RF	AF1	AF2
Annotated in Nr	30,901 (13.65%)	27,843 (34.85%)	28,043 (39.39%)	27,298 (31.80%)
Annotated in Nt	85,294 (37.67%)	53,036 (66.38%)	51,205 (71.93%)	53,220 (62.00%)
Annotated in KO	16,636 (7.35%)	15,288 (19.13%)	15,348 (21.56%)	16,116 (18.77%)
Annotated in Swiss‐Prot	26,122 (11.54%)	25,661 (32.12%)	26,000 (36.52%)	24,866 (28.97%)
Annotated in PFAM	27,231 (12.03%)	18,866 (23.61%)	18,210 (25.58%)	19,069 (22.21%)
Annotated in GO	27,450 (12.12%)	19,047 (23.84%)	18,377 (25.82%)	19,246 (22.42%)
Annotated in KOG	9,040 (3.99%)	9,303 (11.64%)	9,330 (13.11%)	9,014 (10.50%)
Annotated in all Databases	5,926 (2.62%)	5,365 (6.71%)	5,328 (7.48%)	5,803 (6.76%)
Annotated in at least one Database	92,697 (40.94%)	54,455 (68.15%)	52,204 (73.33%)	54,888 (63.94%)
Total Unigenes	226,409	79,900	71,187	85,839

A total of 265,355 (CF: 112,481, RF: 51,661, AF1: 47,323, and AF2: 53,890) CDSs were identified by BLASTX and prediction of the transcripts, and most of them were predicted by Estscan (72.6%–83.6%). To avoid spurious hits, only CDSs longer than 300 nucleotides, CF: 34,974, RF: 23,312, AF1: 22,486, and AF2: 23,490, were annotated for subsequent analysis (Table 4).

**TABLE 4:** Statistics of CDS

Sample	Number of Blast to Protein database		Number of prediction by Estscan	
	Total (Percent)	>300	Total (Percent)	>300
CF	18,476 (16.4%)	13,800	94,005 (83.6%)	21,074
RF	13,333 (25.8%)	10,256	38,328 (74.2%)	13,056
AF1	12,967 (27.4%)	10,013	34,356 (72.6%)	12,473
AF2	14,066 (26.1%)	11,000	39,824 (73.9%)	12,490

Note

>300: Number of CDS longer than 300 nucleotides.

### 3.3 Identification of gene orthologous groups

**FIGURE 3:**
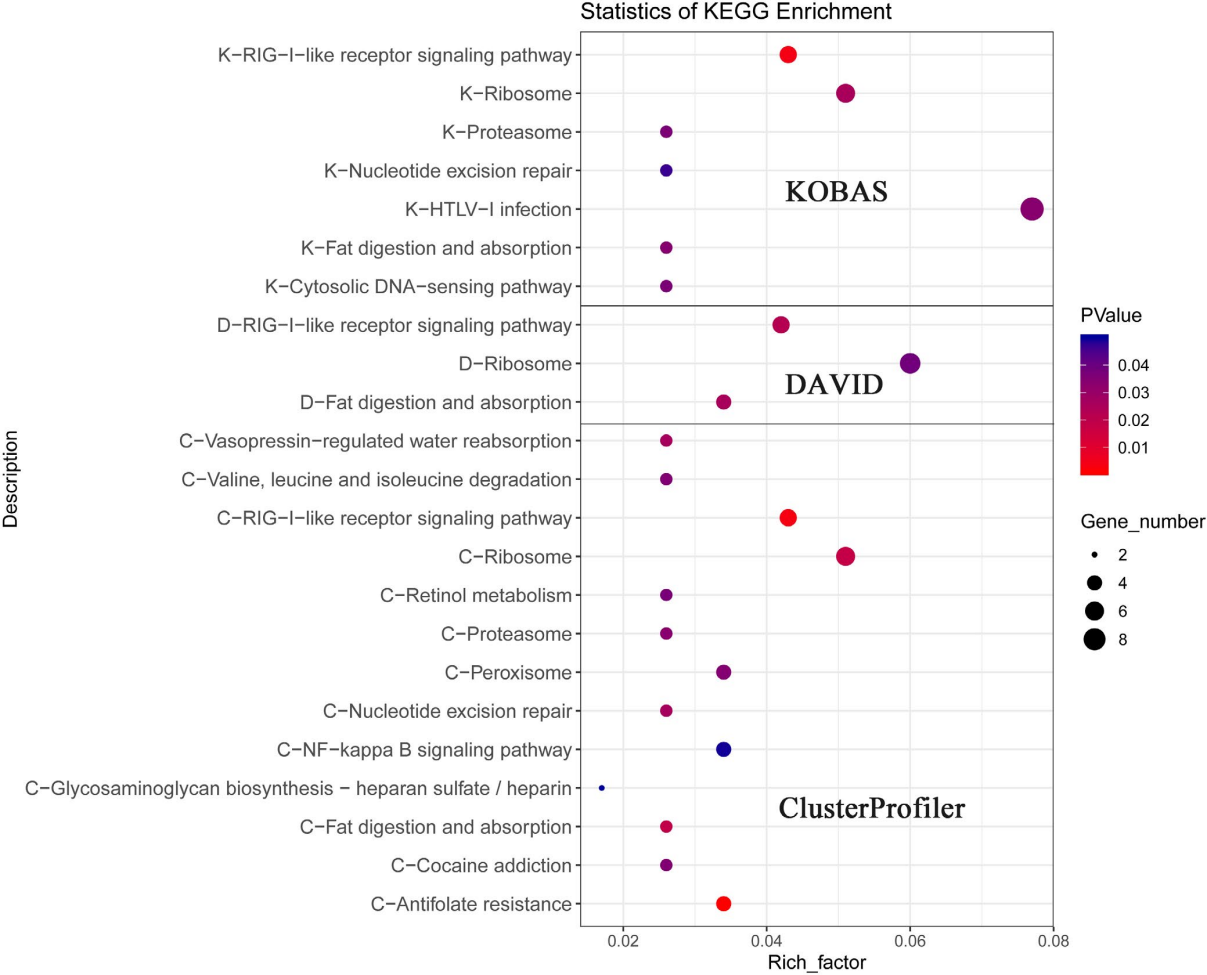
Scatter diagram of KEGG functional classifications for orthologous genes using the KOBAS (K), DAVID (D), and ClusterProfiler (C) methods. The x‐axis shows the percentage of gene number/background of orthologous genes, and the y‐axis shows the enriched KEGG pathway

A total of 21,985 homologous genes were identified in the three species. After filtering, only 393 orthologous genes were selected for the following analysis. Two online software programs (DAVID and KOBAS) and an R package ClusterProfiler were used for enrichment analysis of orthologous genes. Different enrichment methods have been applied in research on the function of orthologous genes. Ultimately, 393 single‐copy genes were annotated in 355 (ClusterProfiler: 149, DAVID: 17, KOBAS: 189, *p*‐value > .05) GO terms and 23 (ClusterProfiler: 13, DAVID: 3, KOBAS: 7, *p*‐value > .05, Figure 3) KEGG pathways.

Of these, several protein‐related terms were identified, such as negative regulation of protein catabolic process (GO:0042177, *p* = .0006), negative regulation of cellular protein catabolic process (GO:1903363, *p* = .0006), protein catabolic process (GO:0030163, *p* = .004), fat digestion and absorption (cfa04975, *p* = .026). Three genes, *AQP3*, *EGR1*, and *FABP1*, were annotated with pathways associated with hypoxic response (cellular response to decreased oxygen levels, GO:0036294, *p* = .044 and cellular response to hypoxia, GO:0071456, *p* = .044). In addition, a large number of genes were also enriched in immune‐related pathways.

### 3.4 Calculation of Ka/Ks to identify positively selected genes

**FIGURE 4:**
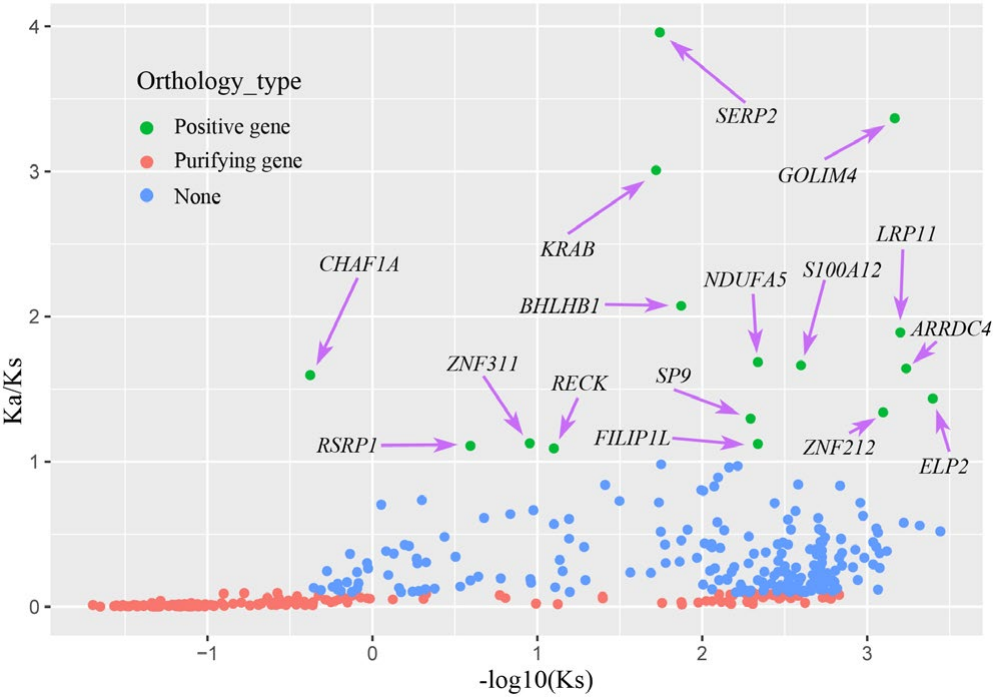
Ka/Ks distribution diagram of genes under positive and purifying selection. The x‐axis shows the value of Ka/Ks. We set the y‐axis to ‐log10(Ks) to highlight the positively selected genes

To reveal the genetic mechanism of adaptation in response to the environment, Paml‐codeml software was used to perform selective stress analysis on the identified single‐copy orthologous genes. The 393 identified single‐copy orthologous genes were used to calculate the Ka/Ks ratio. The results showed that 16 orthologous genes were identified to be under positive selection (*ω* > 1, Table S2, Figure 4).

Enrichment analysis of positive genes showed that the *LRP11* gene was enriched in several pathways related to the temperature response, such as response to cold (GO:0009409, *p* = .087), response to heat (GO:0009408, *p* = .019), and response to temperature stimulus (GO:0009266, *p* = .029). In addition, the *ARRDC4*, *NDUFA5*, and *OLIG2* genes were enriched in pathways related to ubiquitin proteins, respiration, and neuroregulation, respectively.

### 3.5 Homologous gene analysis of three species of fox

**FIGURE 5:**
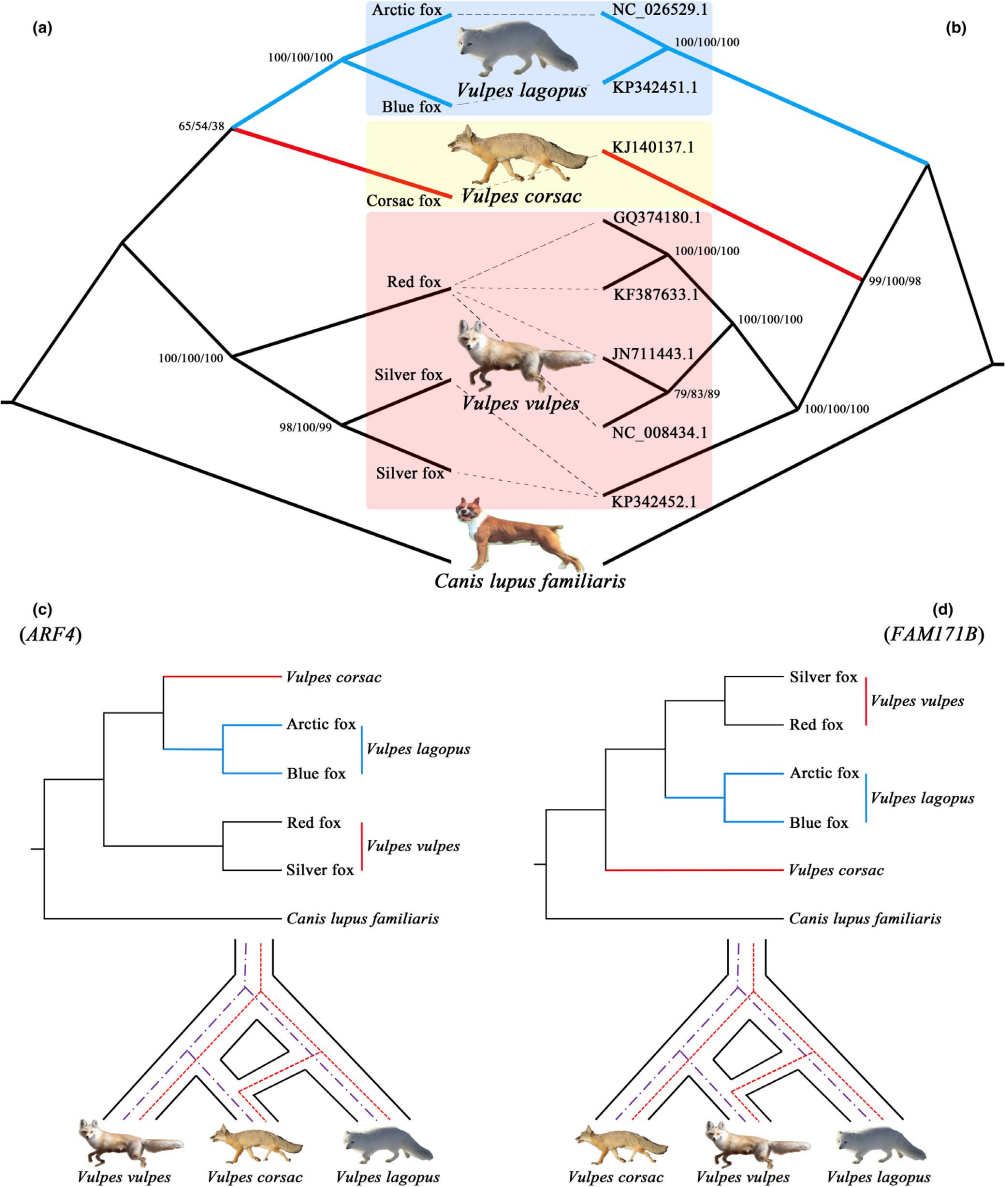
Homologous gene analysis of three species of fox. (a) Phylogenetic tree based on orthologous genes; (b) phylogenetic tree based on mitochondrial DNA. The values at each branch are the posterior probabilities of PAUP, Bayes, and RAxML. (c) and (d) Schematic diagram of the differences between species trees and gene trees. The purple dotted line represents the results of the species tree, and the red dotted line represents the results of the gene tree

Combined with our previously published study, a total of 27 orthologous genes were obtained in five samples. Among them, the *N4BP1* and *TWSG1* genes have sequence deletions in some individuals. It can be concluded that such deletions are caused by sequencing, since the targets of transcriptome sequencing were coding RNA. Therefore, the remaining 25 genes were used for subsequent analysis. Three methods were used to construct ML and BI trees based on 25 genes (Figure 5a) and mitochondrial protein‐coding genes (Table S3, Figure 5b).

As shown in Figure 5, the mitochondrial phylogenetic tree was the same as the traditional classification, but it was different from the results of orthologous gene construction. That is, the classification status of the corsac fox was different. To address this, gene trees of the 25 orthologous genes were constructed using RAxML and analyzed according to previous studies.

A total of 15 gene trees were constructed from 25 orthologous genes (Figure S1). Among them, the gene trees of *ARL3*, *CCDC151*, *GPC3*, *RDH10*, and *TP53I3* were the same as the tree constructed by mitochondria, and of the rest of the evolutionary tree, there were two main types: (1) the gene tree of 9 genes (*ARF4*, *F13B*, *OTUD5*, *RGS4*, *SCNN1B*, *ANAPC5*, *PXN*, *FAM122A*, and *CAMLG*) was the same as the tree constructed by orthologous genes. That is, corsac fox and arctic fox were the first cluster, with rad fox being the second cluster (Figure 5c); (2) the gene tree of 6 genes (*FAM171B*, *MGST1*, *GPLPH3*, *RARRES2*, *RND1*, and *LONRF1*) showed that rad fox and arctic fox were the first cluster, with corsac fox being the second cluster (Figure 5d).

## 4 DISCUSSION

*Vulpes corsac*, *Vulpes vulpes*, and *Vulpes lagopus* have evolved different physiological, morphological, and behavior adaptations to cope with harsh environments. In the present study, comparative transcriptome analysis was used to identify the selected genes and pathways between them.

The results of orthologous gene enrichment showed that three genes (*AQP3*, *EGR1*, and *FABP1*) were annotated with pathways associated with the hypoxic response. The *AQP3* gene encodes aquaporin 3, which is located in the basolateral membrane of renal collecting duct cells and could promote the transport of small non‐ionic solutes such as urea and glycerol (Hou et al., 2016). The *FABP1* gene encodes fatty acid‐binding proteins, which are highly conserved hydrophobic proteins that are responsible for the uptake, transport, and metabolism of fatty acids (Linssen et al., 1990). In addition, there were also a large number of pathways related to nerve cells, immunity, and protein and lipid metabolism, including a large number of genes. These results suggest that these important homologous genes might have been preserved in response to harsh environments.

Selective pressure analysis results showed that a total of 16 genes were screened under positive selection from 393 single‐copy orthologous genes. Enrichment analysis of positive genes showed that the *LRP11* gene which encodes low‐density lipoprotein receptor‐associated protein was enriched in several pathways related to the low‐temperature response. In addition to our enrichment results, previous studies have shown that it is involved in animal anxiety responses (Cai, 2015). We speculated that this gene might play a key role in the response to environmental stimuli of animals, and the positive selection of this gene might be the evolution of foxes to adapt to various environmental changes, such as cold and hunger.

In addition, several genes were related to DNA damage repair (*ELP2* and *CHAF1A*), innate immunity (*ARRDC4* and *S100A12*) and the respiratory chain (*NDUFA5*). Among these genes, the *ARRDC4* gene was involved in a pathway involving ubiquitin proteins. This gene has also been shown to promote *K63* polyubiquitination of *MDA5* through *TRIM65* and regulate the innate immune response induced by enterovirus (Meng et al., 2017). The protein encoded by the *S100A12* gene acts as an inflammatory mediator molecule that activates *RAGE* and TLR‐4 mediated autoimmune responses. It could also affect the transport of some lipoproteins and increase the calcification of blood vessels (Li & Dewey, 2011). Among the remaining positive selection genes, there were three zinc finger protein genes (*ZNF212*, *KRAB*, and *ZNF311*). The main role of *KRAB* is to induce gene silencing and maintain genome stability, which plays a huge role in determining the evolution of species (Ivanov et al., 2007; Rowe & Trono, 2011). Studies on orthologous transcription factors in Drosophila have found that there were widespread differences in the binding of this protein to DNA, and such differences occur in a progressive and evolutionarily feasible way (Siggers et al., 2014). The adaptation of zinc finger proteins combined with the characteristics of new sites enhanced the adaptive evolution of species (Wang et al., 2016). These positively selected genes involved in the stress response, DNA damage repair, and innate immunity might play a role in adaptation to harsh wild fox environments.

The results of common orthologous gene analysis showed that the gene tree constructed by orthologous genes was quite different from the species tree. A literature review shows that the main causes of this situation were horizontal gene transfer (HGT), gene duplication and loss, hybridization, and convergent evolution (Degnan & Rosenberg, 2009). Among the possible reasons, HGT is generally believed to occur in microorganisms, such as bacteria, and does not affect phylogenetic relationships in higher animals (Yang, 2017). Sequence alignment results showed that gene duplication or loss did not occur in these orthologous genes. Thus, we speculate that gene flow or convergent evolution might have contributed to this result. (1) If this difference was due to hybridization, then we hypothesize that there was no reproductive isolation between the three species prior to a certain point in time, so gene flow may have existed between them. This gene flow may be an important factor in promoting regional differentiation of species (Yang, 2017). (2) If these differences were due to convergent evolution, it might mean that these genes evolved convergent in different foxes to adapt to the same environment. In addition, a large number of CDSs discovered in the fox transcriptome data will provide basic transcriptome data for future research.

## 5 CONCLUSIONS

Despite not representing an entire genome, comparative transcriptome analysis was used to identify numerous genes and pathways that are relevant to adaptation in nonmodel organisms. Our study assembled and characterized the first transcriptome data for corsac fox using RNA‐Seq technology and revealed candidate *LRP11* genes that might be involved in cold environment adaptation in foxes. The respiratory chain, innate immunity, and DNA damage repair genes under positive selection might play a role in adaptation to temperature fluctuations and energy expenditure in foxes. In addition, gene flow and convergent evolution might also improve the fitness of foxes. These transcriptomic data will not only promote future studies of evolution and adaptation in foxes and other canids but also provide important resources for the conservation of foxes.

## CONFLICT OF INTEREST

The authors declare that they have no conflicts of interests.

## AUTHOR CONTRIBUTIONS

**Xiufeng Yang:**Conceptualization (equal); Formal analysis (lead); Software (equal); Writing – original draft (lead). **Guolei Sun:** Funding acquisition (equal); Software (equal); Writing – review & editing (lead). **Tian Xia:** Formal analysis (equal); Software (equal). **Muha Cha:** Resources (equal). **Lei Zhang:** Software (equal). **Bo Pang:** Resources (equal). **Qingming Tang:** Resources (equal). **Huashan Dou:** Resources (lead). **Honghai Zhang:** Conceptualization (equal); Funding acquisition (lead).

## ETHICAL APPROVAL

All sample procedures and experimental methods were approved by the Qufu Normal University Institutional Animal Care and Use Committee (No. QFNU2017‐024), Qufu, China.

## Supporting information

Fig S1

Table S1

Table S2

Table S3

## Data Availability

All data analyses during this study are deposited in the Sequence Read Archive database with accession number of SRR16229812. Appendices in the study are available in Dryad (https://doi.org/10.5061/dryad.h9w0vt4kx).
