# Supplementary figures and images for: Transcriptome analysis provides new insights into cold adaptation of corsac fox (Vulpes Corsac)
Source: Ecol Evol. 2022 Apr 19;12(4):e8866. doi: 10.1002/ece3.8866 (PMC9019142; doi:10.1002/ece3.8866)

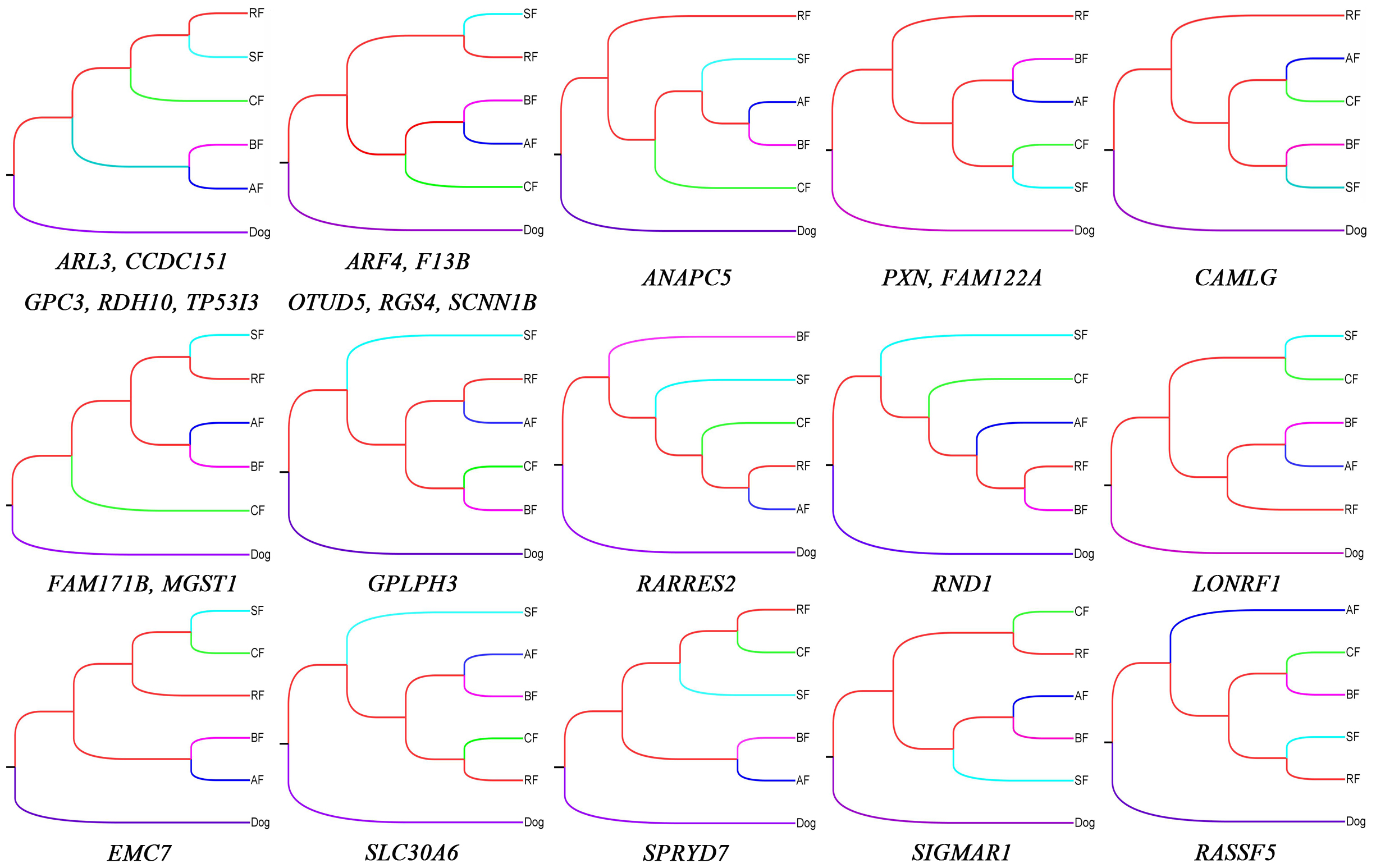

Supplement: Supplementary file 1 — Fig S1 [file ECE3-12-e8866-s001.jpg]
