# Supplementary material for: Transcriptome analysis provides new insights into cold adaptation of corsac fox (Vulpes Corsac)
Source: Ecol Evol. 2022 Apr 19;12(4):e8866. doi: 10.1002/ece3.8866 (PMC9019142; doi:10.1002/ece3.8866)
Supplement: Supplementary file 2 — Table S1 [file ECE3-12-e8866-s002.docx]

Supplementary Table S1 The website and parameters of database.

| Database | Website | Parameter |
| --- | --- | --- |
| Nt | http://www.ncbi.nlm.nih.gov/ | NCBI blast 2.2.28+ |
|  |  | e-value = 1e-5 |
| Nr | http://www.ncbi.nlm.nih.gov/ | diamond v0.8.22 |
|  |  | e-value = 1e-5, --more-sensitive |
| KEGG | http://www.genome.jp/kegg/ | KAAS，KEGG Automatic Annotation Server  e-value= 1e-10 |
| Swiss-Prot | http://www.ebi.ac.uk/uniprot/ | diamond v0.8.22 |
|  |  | e-value = 1e-5, --more-sensitive |
| PFAM | http://pfam.sanger.ac.uk/ | HMMER 3.0 package，hmmscan |
|  |  | e-value = 0.01 |
| GO | http://www.geneontology.org/ | e-value = 1e-6 |
| KOG/COG | http://www.ncbi.nlm.nih.gov/COG/ | diamond v0.8.22 |
|  |  | e-value = 1e-3, --more-sensitive |
