# Supplementary material for: Transcriptome analysis provides new insights into cold adaptation of corsac fox (Vulpes Corsac)
Source: Ecol Evol. 2022 Apr 19;12(4):e8866. doi: 10.1002/ece3.8866 (PMC9019142; doi:10.1002/ece3.8866)
Supplement: Supplementary file 3 — Table S2 [file ECE3-12-e8866-s003.docx]

Supplementary Table S2 Positive selection gene

| Gene ID | Gene name | Ka/Ks | Ka | Ks |
| --- | --- | --- | --- | --- |
| *FILIP1L* | Filamin A interacting protein 1 like | 1.12259 | 0.00514 | 0.0046 |
| *ARRDC4* | Arrestin domain containing 4 | 1.64169 | 0.00096 | 0.00058 |
| *RSRP1* | Arginine and serine rich protein 1 | 1.10914 | 0.28228 | 0.25448 |
| *S100A12* | S100 calcium binding protein A12 | 1.6637 | 0.0042 | 0.00252 |
| *ELP2* | Elongator acetyltransferase complex subunit 2 | 1.43446 | 0.00056 | 0.0004 |
| *CHAF1A* | Chromatin assembly factor 1 subunit A | 1.59571 | 3.80222 | 2.38278 |
| *GOLIM4* | Golgi integral membrane protein 4 | 3.36629 | 0.0023 | 0.00068 |
| *ZNF212* | Zinc finger protein 212 | 1.33937 | 0.00106 | 0.0008 |
| *NDUFA5* | NADH:ubiquinone oxidoreductase subunit A5 | 1.67954 | 0.00748 | 0.00446 |
| *LRP11* | LDL receptor related protein 11 | 1.88368 | 0.00114 | 0.0006 |
| *KRAB* | ZFP57 zinc finger protein | 3.00846 | 0.05738 | 0.01906 |
| *OLIG2* | Oligodendrocyte transcription factor 2 | 2.07382 | 0.02784 | 0.01342 |
| *SP9* | Sp9 transcription factor | 1.3141 | 0.00666 | 0.00508 |
| *SERP2* | Stress associated endoplasmic reticulum protein family member 2 | 3.958 | 0.07156 | 0.01808 |
| *ZNF311* | Zinc finger protein 311 | 1.12701 | 0.12528 | 0.11114 |
| *RECK* | Reversion inducing cysteine rich protein with kazal motifs | 1.09088 | 0.08666 | 0.07942 |
