# Supplementary material for: Transcriptome analysis provides new insights into cold adaptation of corsac fox (Vulpes Corsac)
Source: Ecol Evol. 2022 Apr 19;12(4):e8866. doi: 10.1002/ece3.8866 (PMC9019142; doi:10.1002/ece3.8866)
Supplement: Supplementary file 4 — Table S3 [file ECE3-12-e8866-s004.docx]

Supplementary Table S3 Mitochondrial sequence information for tree building

| Taxon Name | Common Name | Accession Number |
| --- | --- | --- |
| *Vulpes lagopus* | Arctic fox | NC_026529.1 |
|  | Blue fox | KP342451.1 |
| *Vulpes vulpes* | Red fox | GQ374180.1 |
|  |  | KF387633.1 |
|  |  | JN711443.1 |
|  |  | NC_008434.1 |
|  | Silver fox | KP342452.1 |
| *Vulpes corsac* | Corsac fox | KJ140137.1 |
| *Canis lupus familiaris* | Dog | NC_002008.4 |
